# Association between Social Relationship and Glycemic Control among Older Japanese: JAGES Cross-Sectional Study

**DOI:** 10.1371/journal.pone.0169904

**Published:** 2017-01-06

**Authors:** Kenichi Yokobayashi, Ichiro Kawachi, Katsunori Kondo, Naoki Kondo, Yuiko Nagamine, Yukako Tani, Kokoro Shirai, Susumu Tazuma, JAGES group

**Affiliations:** 1 Department of Social and Behavioral Sciences, Harvard T.H. Chan School of Public Health, Boston, MA, United States of America; 2 Department of General Internal Medicine, Hiroshima University of Medical Science, Hiroshima, Japan; 3 Center for Preventive Medical Sciences, Chiba University, Chiba, Japan; 4 Department of Public Health, Chiba University Graduate School of Medicine, Chiba, Japan; 5 Department of Gerontology and Evaluation Study, Center for Gerontology and Social Science, National Center for Geriatrics and Gerontology, Obu city, Aichi, Japan; 6 Department of Health Economics and Epidemiology Research, The University of Tokyo, Tokyo, Japan; 7 Department of Epidemiology and Public Health, University College London, London, United Kingdom; 8 Department of Global Health Promotion, Tokyo Medical and Dental University, Tokyo, Japan; 9 Research Fellow of Japan Society for the Promotion of Science, Tokyo, Japan; 10 Department of Human Sciences, School of Law and Letters, University of the Ryukyus, Okinawa, Japan; Hamamatsu Ika Daigaku, JAPAN

## Abstract

**Aim:**

The present study examined whether social support, informal socializing and social participation are associated with glycemic control in older people.

**Methods:**

Data for this population-based cross-sectional study was obtained from the Japan Gerontological Evaluation Study (JAGES) 2010 linked to the annual health check-up data in Japan. We analyzed 9,554 individuals aged ≥65 years without the certification of needed long-term care. Multivariate logistic regression models were used to assess the effect of social support, informal socializing and social participations on glycemic control. The outcome measure was HbA1c ≥8.4%.

**Results:**

1.3% of the participants had a level of HbA1c over 8.4%. Better glycemic control was significantly associated with meeting with friends one to four times per month (odds ratio [OR] 0.51, 95% confidence interval [CI]0.30–0.89, compared to meeting with friends a few times per year or less) and participation in sports groups (OR 0.50, 95% CI 0.26–0.97) even after adjusting for other variables. Meeting with friends more than twice per week, receiving social support, and being married were not associated with better control of diabetes.

**Conclusions:**

Meeting with friends occasionally is associated with better glycemic control among older people.

## Introduction

Diabetes is among the most common chronic diseases, affecting 347 million people worldwide [1]. Diabetes has become one of the biggest cause of premature death in many countries. Total deaths from diabetes are going to rise by more than 50% in the next 10 years [1]. Japan ranks in the top ten countries with regard to the number of diabetic patients in the population [2] and the prevalence of diabetes stands at 16.2% in men and 9.2% in women [3]. To prevent diabetic complications, the Japanese government has issued a target to reduce the number of diabetic patients with HbA1c levels of over 8.4% [4]. However, glycemic control can be difficult to achieve in older patients because of difficulties in adhering to detailed diet and exercise instructions, as well as the challenges of using prescribed antidiabetic agents due to the risk of low blood sugar [5].

Previous studies have shown that social relationships such as social networks and support are associated with better disease management outcomes. Studies have shown that social relationships affect diabetic health outcomes such as quality of life, mental health and glycemic control in a positive way [6][7]. However, most studies were based on adult diabetes outpatients and were not specifically focused on older people. In addition, no study has directly compared the influence of social support, informal socializing, and social participation on glycemic control in older people.

The term “social support” must be distinguished from “social network.”[6] Social support is an interpersonal psychosocial resource. It is an individual’s perception that one is accepted or cared for. Social support can arise from various sources such as friends, family and healthcare providers [7]. On the other hand, social networks are based on the existence of relationships (e.g., the number of one’s friends, the frequency of contact) [7][8]. Informal socializing—such as frequency of meeting with one’s friends—is considered a structural indicator of network contacts. Social participation means participation in community organizations [9].

The purpose of this study was to investigate whether social support, informal socializing and social participation are associated with improved glycemic control in older people.

## Materials and Methods

### Study sample

Data for this study was obtained from the Japan Gerontological Evaluation Study (JAGES) [10][11] linked to the annual health check-up data in Japan. The JAGES was established in 2010 to investigate the social determinants of health among community-dwelling individuals aged 65 years or older without the certification of needed long-term care, that is, who did not already have physical or cognitive disabilities. The survey covered 31 municipalities in 12 of 47 prefectures in Japan. In this study, we used the JAGES data from Aichi prefecture where we managed to link participant data with annual health checkup data (maintained by local public health authorities). In 2010, self-administered questionnaires were mailed to 33,818 residents in six municipalities in Aichi prefecture (Tokoname city, Toukai city, Chita city, Higashiura town, Minamichita town, and Taketoyo town). The targeted municipalities ranged from urban, semi-urban, and rural settings. The baseline survey randomly sampled one of every four residents in the 3 large municipalities (Toukai city, Chita city, and Higashiura town) and obtained a complete census of the residents in the 3 smaller municipalities (Tokoname city, Minamichita town, and Taketoyo town). The data of 20,432 respondents (response rate, 60.4%) was then linked to the annual health check-up data. In Japan, citizens insured under the national health insurance system are required to present to local government clinics for an annual health check-up in order to detect early stage life-style related diseases. The annual health examination includes a medical history, physical examination (including weight and waist circumference), urine and blood tests. Although mandated by the public health law, compliance with annual health checkup visits is typically 38 percent nationally [12].

In the present study, we linked the 20,432 JAGES participant data to the health check-up data, maintained by the local health authorities. A total of 9,893 cohort participants were successfully linked to their health check-up data. We analyzed 9,554 subjects, excluding 339 people with missing values for HbA1c.

### Outcome measures (HbA1c)

The outcome measure was HbA1c≥8.4% (NGSP) measured by latex coagulating method, considered to be the cutoff for satisfactory glycemic control according to Japanese government targets [4].

### Primary explanatory variables (social support, informal socializing and social participation)

We collected survey measures of emotional and instrumental support (both providing and receiving), as indicators of social support. For example, receiving emotional social support was assessed by asking the following question ‘Do you have someone who listens to your concerns and complaints?’ while providing instrumental support to others was evaluated using the question ‘Do you have someone you take care of when she/he is sick in bed?’ These were dichotomized into yes and no. Responses of “none” to these questions were defined as no [13]. Frequency of meeting with friends was the variable used to measure informal socializing. It was categorized as more than twice per week (high frequency), one to four times per month (middle frequency) and a few times per year or less (low frequency). Social participation was classified into eight types of groups: political organizations, industrial or trade associations, volunteer groups, senior citizens clubs, religious organizations, sports groups or clubs, neighborhood associations, and hobby groups. Subjects were dichotomized based on the frequency of their participation in these groups: more than once per month (high participation) versus a few times per year or less (low participation).

### Adjustment variables

Sex and age group were used as demographic covariates. Because physiological, psychological, socioeconomic and behavioral pathways may be potential mechanisms for social relationships to influence health [14], these factors were obtained. To examine the effect of physiological effects, information was included on body mass index (BMI), which was divided into four categories (underweight: <18.5; normal weight: 18.5–24.9; overweight: ≥25), and walking time per day (<30 min, 30–89 min, ≥90 min). For psychological status, we used Geriatric Depression Scale (GDS) which was divided into two categories, that is, non-depressed (0–4) and depressed (5–15). used. Socioeconomic status was assessed using equivalized household income (low: <2.0 million yen; high: ≥2.0 million yen), educational attainment (low: ≤9 years; high: ≥10 years), marital status (married, single, others), eating conditions (eating with others, eating alone) and living status (living with others, living alone). For health behavior, we asked smoking status (never, have stopped smoking, current smoke) and the frequency of alcohol consumption (never, have stopped, current drinking). Self-reported diabetes treatment condition was also included in the multivariable models.

### Statistical analysis

We used logistic regression analysis to estimate the ORs of the social relationships and other potential confounding factors for glycemic control. In order to evaluate the independent association of social support, informal socializing and social participation, we separately analyzed those three social relationship variables with all other adjustment variables (Model 1). In Model 2, all three social relationships were investigated simultaneously with all covariates. We also examined the interaction effect of informal socializing and educational status because educational attainment might be associated with frequency of social interaction among diabetics [15]. We used dummy variables to create missing categories for independent variables with missing values.

All statistical analyses were carried out using STATA 12 (StataCorp. 2011. Stata Statistical Software: Release 12. College Station, TX: StataCorp LP). The level of significance was established at *P* < 0.05 (two sided) for all tests.

### Ethics committee

This study was approved by the Nihon Fukushi University Ethics Committee (No. 10–05). The voluntary return of the self-administered questionnaire was interpreted as informed consent.

## Results

The mean age (standard deviation) was 72.7 (5.6) years. 1.3% of the participants had a level of HbA1c over 8.4% (Table 1). Among those who were under diabetes treatment with 11.1%, 9.1% of them had a level of HbA1c over 8.4%.

**Table 1:** Baseline characteristics.

	n	%
**Sex**		
Female	5,159	54.3
Male	4,359	45.7
**Age (years)**		
65–69	3,312	34.6
70–74	3,151	33.0
75–79	1,832	19.2
80–84	912	9.6
≥85	347	3.6
**HbA1c (mg/dL)**		
≥8.4	120	1.3
<8.4	9,434	98.7
***Social support***		
**Receiving emotional support**		
Yes	8,541	89.4
No	483	5.1
Missing	530	5.5
**Providing emotional support**		
Yes	8,360	87.5
No	535	5.6
Missing	659	6.9
**Receiving instrumental support**		
Yes	8,632	90.4
No	402	4.2
Missing	520	5.4
**Providing instrumental support**		
Yes	8,006	83.8
No	793	8.3
Missing	755	7.9
***Informal socializing***		
**Frequency of meeting with friends**		
High (twice or more / week)	3,657	38.3
Middle (one to four times / month)	3,386	35.4
Low (a few times a year or less)	1,868	19.6
Missing	643	6.7
***Social participation***		
**Politics**		
High participation	362	3.8
Low participation	7,033	73.6
Missing	2,159	22.6
**Industry**		
High participation	385	3.0
Low participation	6,821	71.4
Missing	2,348	24.6
**Volunteering**		
High participation	1,045	10.9
Low participation	6,324	66.2
Missing	2,185	22.9
**Senior citizen club**		
High participation	1,445	15.1
Low participation	6,379	66.8
Missing	1,730	18.1
**Religion**		
High participation	572	6.0
Low participation	6,997	73.2
Missing	1,985	20.8
**Sports**		
High participation	2,156	22.6
Low participation	5,627	58.9
Missing	1,771	18.5
**Neighborhood community**		
High participation	771	8.1
Low participation	6,903	72.3
Missing	1,880	19.7
**Hobby**		
High participation	3,419	35.8
Low participation	4,566	47.8
Missing	1,569	16.4
**BMI (kg/m**^**2**^**)**		
Underweight (<18.5)	622	6.5
Normal (18.5–24.9)	6,665	69.8
Overweight (≥25.0)	2,267	23.7
**Depression (GDS score)**		
Non-depressed (<5)	5,881	61.6
Depressed (≥5)	2,012	21.1
Missing	1,661	17.4
**Equivalent household income (million yen)**		
Low (<2.0)	3,873	40.5
High (≥2.0)	3,909	40.9
Missing	1,772	18.6
**Education (years)**		
Low (≤9)	4,376	45.8
High (≥10)	4,704	49.2
Other/missing	474	5.0
**Walking time (per day)**		
<30 min	2,792	29.2
30–89 min	4,709	49.3
≥90 min	1,459	15.3
Missing	594	6.2
**Marital status**		
Married	7,077	74.1
Single	2,048	21.4
Other	31	0.3
Missing	398	4.2
**Eating conditions**		
Eating with others	7,189	75.2
Eating alone	1,811	20.0
Missing	554	5.8
**Living status**		
Living with others	8,545	89.4
Living alone	1,009	10.6
**Smoking**		
Never	5,299	55.5
Have stopped	2,467	25.8
Current	814	8.5
Missing	974	10.2
**Alchohol**		
Never	5,307	55.6
Have stopped	274	2.9
Current	3,336	34.9
Missing	637	6.7
**Diabetes treatment**		
Yes (under treatment)	1,058	11.1
No	8,271	86.6
Missing	225	2.3

Table 2 shows the associations between glycemic control and social relationships (social support, informal socializing and social participation). The odds ratio (OR) for poorly-controlled diabetes for those who met with friends one to four times per month compared with those who met with friends a few times a year or less was 0.52 (95% CI: 0.29–0.93, Model 2). In contrast, the OR for those who met with friends more than twice per week compared with those who met with friends rarely was 0.76 (95% CI: 0.43–1.34, Model 2). This pattern for the association between glycemic control and frequency of meeting with friends was similar across crude model, Model 1 and Model 2. Having no friends was associated with poorly-controlled diabetes (OR: 3.90, 95%CI: 1.65–9.20). Among the different types of social participation, participating in sports clubs showed a significant protective association with glycemic control (OR: 0.50, 95%CI: 0.25–0.97, Model 2). Social support was not associated with poorly-controlled diabetes.

**Table 2:** Odds ratios of social relationships on glycemic control (HbA1c ≥8.4 vs <8.4).

		n of ≥8.4	Crude	Model 1	Model 2
			OR (95% CI)	OR (95% CI)	OR (95% CI)
**Informal socializing (frequency of meeting with friends)**					
Low		32	ref	ref	ref
Middle		29	0.45 (0.27–0.76)	0.51 (0.30–0.89)	0.52 (0.29–0.93)
High		45	0.64 (0.41–1.03)	0.68 (0.41–1.14)	0.76 (0.43–1.34)
**Social support (ref: no)**					
Receiving emotional support		103	0.43 (0.13–1.39)	0.35 (0.10–1.23)	0.31 (0.09–1.12)
Providing emotional support		96	2.03 (0.54–5.37)	1.97 (0.79–4.87)	1.83 (0.72–4.63)
Receiving instrumental support		106	0.94 (0.65–4.74)	1.29 (0.43–3.83)	1.42 (0.47–4.29)
Providing instrumental support		91	1.33 (0.67–2.64)	1.28 (0.59–2.75)	1.27 (0.58–2.77)
**Social participation (ref: low participation)**					
Politics		5	1.09 (0.38–3.16)	1.20 (0.37–3.86)	1.14 (0.35–3.67)
Industry		4	1.09 (0.37–3.23)	1.14 (0.36–3.58)	1.36 (0.42–4.35)
Volunteering		11	0.85 (0.41–1.76)	0.94 (0.43–2.05)	0.93 (0.42–2.06)
Senior citizen club		13	0.79 (0.40–1.55)	0.84 (0.41–1.74)	0.88 (0.42–1.82)
Religion		5	0.72 (0.27–1.89)	0.63 (0.23–1.71)	0.62 (0.23–1.71)
Sports		17	0.54 (0.30–0.99)	0.50 (0.26–0.97)	0.50 (0.25–0.97)
Neighborhood community		12	1.79 (0.92–3.50)	1.62 (0.78–3.37)	1.63 (0.78–3.42)
Hobby		34	0.84 (0.51–1.38)	0.83 (0.48–1.43)	0.90 (0.51–1.58)

OR, odds ratio; 95% CI, 95% confidence intervals; ref, reference.

All models were adjusted for age, sex, BMI, GDS, household income, educational level, walking time per day, marital status, eating and living status, diabetes treatment condition, smoking and alcohol status

People who did not finish high school were more likely to have better glycemic control when they met with their friends sometimes to frequently (Table 3). For men, better glycemic control was significantly associated with meeting with friends one to four times per month (Table 4). There was no association between married and single people in terms of informal socializing and glycemic control (OR: 0.74, 95%CI: 0.40–1.39). Eating alone compared with eating with others was not associated with glycemic control (OR: 0.89, 95%CI: 0.42–1.84). Living alone compared with living with others was not significantly related to glycemic control (OR: 1.13, 95%CI: 0.43–1.84).

**Table 3:** Adjusted odds ratios of informal socializing on glycemic control by educational status (HbA1c ≥8.4 vs <8.4).

	Educational status			
	Over 10 years		0–9 years	
	N	OR (95% CI)	N	OR (95% CI)
**Frequency of meeting with friends**				
**Low**	862	ref	920	ref
**Middle**	1,687	0.65 (0.29–1.48)	1,578	0.42 (0.19–0.90)
**High**	1,609	0.99 (0.47–2.08)	1,887	0.49 (0.23–1.03)

Adjusted for age, sex, BMI, GDS, household income, educational level, walking time per day, marital status, eating and living status, diabetes treatment condition, smoking and alcohol status.

**Table 4:** Adjusted odds ratios of informal socializing on glycemic control by sex (HbA1c ≥8.4 vs <8.4).

	Sex			
	Male		Female	
	N	OR (95% CI)	N	OR (95% CI)
**Frequency of meeting with friends**				
**Low**	1,208	ref	660	ref
**Middle**	1,435	0.42 (0.19–0.92)	1.951	0.62 (0.26–1.47)
**High**	1,468	0.70 (0.36–1.37)	2,189	0.72 (0.30–1.70)

Adjusted for age, sex, BMI, GDS, household income, educational level, walking time per day, marital status, eating and living status, diabetes treatment condition, smoking and alcohol status.

## Discussion

This cross sectional study investigated the association between glycemic control and social relationships (social support, informal socializing and social participation) among older people. To our knowledge, this is the first study comparing directly between different aspects of social relationships and glycemic control among older individuals. We found that meeting with friends one to four times per month and participation in sports groups or clubs were associated with glycemic control even after adjusting for other covariates. On the other hand, meeting with friends more than twice per week, social support, and marital status were not associated with poor glycemic control. Informal socializing was associated with glycemic control among those whose education level was low and among men.

Previous studies have suggested that social relationships affected better glycemic control in patients with diabetes [6][7], but less is known about whether informal socializing is associated with glycemic control. Although studies demonstrating the relationship between the size of the social network and diabetes risk have been conducted [6][7][16], there has not been a study investigating the association between social contact frequency and HbA1c levels. The present study suggests that there is an optimal frequency of meeting with friends (one to four times per month) that may contribute to better glycemic control. More frequent socializing was not associated with improved glycemic control. When the HbA1c cut-off point was shifted from 8.4 to 6.5, meeting with friends one to four times per month tended to be associated with glycemic control (OR 0.87, 95% CI 0.73–1.04).

There may be several possible reasons for the J-shaped relationship. First, meeting with friends on a very frequent basis may involve more occasions for eating and drinking which could lead to glycemic control problems. A previous study showed that eating with friends led to taking larger calories and longer eating time compared with eating alone [17]. Another study showed that there was an association between social support and heavier drinking and higher fat intake [18]. Not moderate but very frequent socializing may lead to taking more calories. Second, a higher frequency of contact with friends can sometimes be associated with a greater obligation to help others, possibly resulting in higher burden and stress [19][20]. Lastly, if people suffer from severe diabetes, their friends could be checking in on them more frequently because of concern about their health. In this case, the diabetic people do not go out to meet with their friends, but instead, the friends come see the patients. Patients who have higher values for HbA1c did report receiving more support from their social network [21].

On the positive side, occasional social interactions appeared to be beneficial for glycemic control. The possible mechanisms include exchange of information about disease management among friends. In support of this hypothesis, the benefit of social interactions was observed mainly among people with lower level of educational attainment, whereas no association was observed among those with higher levels of education. An alternative mechanism is that meeting with friends might involve seniors in more physically active lifestyles, although when we adjusted for self-reported walking time, there was no association between glycemic control and walking. Lastly, we cannot exclude the possibility of reverse causality, i.e. individuals with good glycemic control are more able to meet with their friends frequently.

In our study, better glycemic control was associated with informal socializing among only men. This is opposite of the previous study that men with higher social support appeared to engage in heavier drinking and also reported a higher fat intake pattern [18]. The possible reason for the discrepancy is that the target of the previous study was people aged 40–69 years and men in this age group tend to drink more and consume more high calorie foods while engaging in “nomikai” or “enkai” (after work social gatherings) while socializing with other male colleagues. In contrast, there are fewer opportunities for such social drinking occasions after retirement; therefore informal socializing may have a more positive effect for older people. For women, no significant association between diabetes control and informal socializing was found, which is consistent with the previous study that found no association between social support and metabolic syndrome among women [18].

Social support was not associated with poorly-controlled diabetes in this study. Some previous studies suggested that higher levels of social support were related to improved HbA1c [22][23][24][25][26], but other studies were not able to show a significant relationship between social support and glycemic control [27][28]. Another study found that having high emotional and informational social support led to controlling their diabetes effectively [29]. Although it was not statistically significant, our study also showed that there was a positive association between receiving emotional support and glycemic control. Unfortunately, the participants were not asked specifically about informational support which might be a major factor affecting glycemic control.

Among types of social participation, participating in sports clubs showed a significant positive impact on glycemic control. Since diet and exercise is a main axis for diabetes prevention and management, involvement in sports likely contributes to good glycemic control. On the other hand, high levels of HbA1c may restrict people from sports participation [30]. Since participation in other groups were not associated with glycemic control, we suppose that not social participation but playing sports may have a beneficial effect on glycemic control.

This study had several limitations. First, since this was a cross sectional study, we were not able to infer causality. Moreover, the quality of meeting with friends were not considered. Further prospective and intervention studies are required. Second, there might be a selection bias because the participants were those who received medical checkups. Older people who received checkups were more likely to have social support and participate in social activities [31]. Although the average annual health check-up rate in 2010 among those aged ≥65 years was about 34% in Japan and 38% in the study site [32], 65% of the survey respondents stated that they underwent health check-ups within one year in the questionnaire. Moreover, participants were those without the certification of needed long-term care. Excluded people such as having physical disability by stroke sequela might have higher glycemic value. Finally, we did not consider the comorbidity which may be a confounder.

In conclusion, our population-based, cross-sectional study is the first to suggest that meeting with friends occasionally may benefit glycemic control among older people. Given that achieving glycemic control can be difficult in older patients because of difficulties in understanding detailed dietary and exercise instructions (as well as the challenges of using antidiabetic agents due to the risk of low blood sugar), promoting sociability may be a viable option to assist older diabetic patients in managing their condition. Future longitudinal studies are needed to elucidate the causal associations and identify what kind of socializing is effective.
